# Supplementary material for: Synergistic Combination of AS101 and Azidothymidine against Clinical Isolates of Carbapenem-Resistant Klebsiella pneumoniae
Source: Pathogens. 2021 Nov 29;10(12):1552. doi: 10.3390/pathogens10121552 (PMC8706163; doi:10.3390/pathogens10121552)
Supplement: Supplementary file 1 [file pathogens-10-01552-s001.zip › pathogens-1453738-SI.pdf]

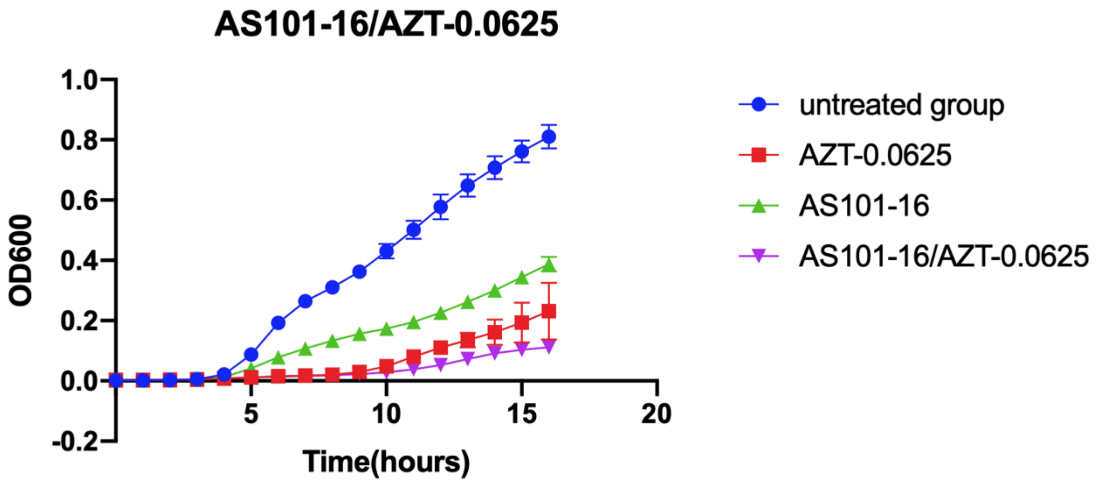

Figure S1: Growth curve of CRE-1044 in synergestic concentration (16μg/mL AS101, 0.0625μg/mL AZT and 16μg/mL AS101- 0.0625μg/mL AZT in combination)
